# One photon-per-bit receiver using near-noiseless phase-sensitive amplification

**DOI:** 10.1038/s41377-020-00389-2

**Published:** 2020-09-02

**Authors:** Ravikiran Kakarla, Jochen Schröder, Peter A. Andrekson

**Affiliations:** Photonics Laboratory, Department of Microtechnology and Nanoscience (MC2), Chalmers University of Technology, 412-96 Gothenburg, Sweden

**Keywords:** Fibre optics and optical communications, Nonlinear optics

## Abstract

Space communication for deep-space missions, inter-satellite data transfer and Earth monitoring requires high-speed data connectivity. The reach is fundamentally dictated by the available transmission power, the aperture size, and the receiver sensitivity. A transition from radio-frequency links to optical links is now seriously being considered, as this greatly reduces the channel loss caused by diffraction. A widely studied approach uses power-efficient formats along with nanowire-based photon-counting receivers cooled to a few Kelvins operating at speeds below 1 Gb/s. However, to achieve the multi-Gb/s data rates that will be required in the future, systems relying on pre-amplified receivers together with advanced signal generation and processing techniques from fibre communications are also considered. The sensitivity of such systems is largely determined by the noise figure (NF) of the pre-amplifier, which is theoretically 3 dB for almost all amplifiers. Phase-sensitive optical amplifiers (PSAs) with their uniquely low NF of 0 dB promise to provide the best possible sensitivity for Gb/s-rate long-haul free-space links. Here, we demonstrate a novel approach using a PSA-based receiver in a free-space transmission experiment with an unprecedented bit-error-free, black-box sensitivity of 1 photon-per-information-bit (PPB) at an information rate of 10.5 Gb/s. The system adopts a simple modulation format (quadrature-phase-shift keying, QPSK), standard digital signal processing for signal recovery and forward-error correction and is straightforwardly scalable to higher data rates.

## Space communication: Opening optical links

Communication links for deep-space exploration spacecraft and satellites could become more efficient using an optical system which can reduce signal losses during transmission and delivers one bit of data per each received photon at a rate of 10 gigabits per second. Peter Andrekson and colleagues at Chalmers University of Technology in Sweden developed the system and demonstrated its potential in laboratory scale experiments. It relies on a technology known as phase-sensitive optical amplification. The researchers transmitted signals across only a one meter, but they believe their work proves the validity of a process that could readily be scaled up for communication across space. Replacing current radio-frequency technology with more effective optical systems could meet the demands of future space communications systems, which will need to operate at higher data rates and across greater distances.

## Introduction

Increased space exploration and the growing capability and thus data output of satellite-borne sensors operated by agencies such as NASA, ESA and JAXA impose greater demands on communication systems to operate at higher data rates and to reach across farther distances into space^1^. Improving the receiver sensitivity is considered the most important method to improve the data throughput with as few photons received as possible. A better receiver sensitivity translates to a longer reach, higher data throughput and the ability to use more compact optics or a combination of the above. Common approaches to improve the sensitivity are known to suffer from a low spectral efficiency (SE) (bits/s per Hz) and only achieve modest net data rates due to their inherent trade-off between sensitivity and bandwidth^2^. In particular, pulse position modulation (PPM) is widely considered in space communications because it reaches an excellent sensitivity at very low signal-to-noise ratios (SNRs)^3^ but experiences a major loss in SE. Photon counting receivers are often employed to receive PPM symbols enabling sensitivities of a few photons per bit. Superconducting nanowire-based versions of these receivers have recently been shown to provide excellent performance, including a quantum efficiency of 90% at data rates up to a few 100 Mb/s^4–6^. A key drawback is, however, the need to be cooled to 2–4 K and their inability to detect photons at rates of multiple Gb/s^7^. Although free space possesses an unrestricted optical bandwidth, the limited detection bandwidth of photon counting receivers restricts the achievable data rates, especially when applying higher-order PPM, which is spectrally very inefficient.

The demonstrated record sensitivities of photon counting receivers with PPM modulation include a sensitivity of ~1 photon-per-information-bit (PPB) at 14 Mbps^8^ and 1.2 PPB at 38 Mbps^9^. However, the low efficiencies of photon counting receivers at high frequencies result in a relatively modest performance at high data rates. The successful application of PPM and photon counting technologies at rates above 100 Mbps has been demonstrated by NASA at 622 Mbps with a 3.8-PPB sensitivity in the Lunar Laser Communication Demonstration (LLCD)^9^ and at 781 Mbps with a sensitivity of 0.5 detected PPB. However, when accounting for the insertion loss and non-ideal quantum efficiency, the latter result corresponds to a black-box sensitivity of ~8 incident PPBs^10^.

Future space communication systems such as inter-satellite and satellite-to-ground links are expected to operate at speeds of several tens of Gb/s and beyond and will thus require a major improvement over existing receiver technology both in terms of data rate and sensitivity^6^. Space communication research has therefore started to adopt technology from the optical fibre communication field, and advanced modulation formats with optically pre-amplified coherent detection in combination with advanced forward error correction (FEC) are a promising solution to improve both the data rate and receiver sensitivity. One impressive result using a single-quadrature (SQ) homodyne detection receiver without a pre-amplifier resulted in a sensitivity of 1.5 PPB at 156 Mb/s^11^, where the data rate was limited by the optical phase-locked loop (PLL) bandwidth. Demonstrations with erbium-doped fibre amplifier (EDFA) pre-amplified coherent receivers resulted in sensitives as low as 2.1 PPB at 10 Gb/s^12–14^.

The theoretically minimal sensitivities are found from inspecting the fundamental capacity limits. The capacity, i.e., the maximum information rate under error-free data transmission for a pre-amplified dual-quadrature or phase-diverse coherent homodyne receiver is^15^ (please refer to the Supplementary Material for the derivation):

1 $${\it{C}}_{{\mathrm{preamp}}} = {\it{B}}{\rm {log}}_2\left( {1 + \frac{{2{\it{S}}}}{{F_{\rm{n}}h\nu B}}} \right)$$

where *F*_n_ is the noise figure (NF) of the pre-amplifier, *S* is the signal power, *h* is Planck’s constant, *ν* is the frequency of the optical carrier wave, and the bandwidth *B* is the inverse of the symbol period. By rewriting *S* = *n*_s_*hvB*, where *n*_s_ is the number of photons per transmitted symbol, the capacity of a pre-amplified receiver becomes *C*_preamp_ = *B*log_2_(1 + 2*n*_s_/*F*_N_) with 2*n*_s_/*F*_N_ interpreted as the SNR of the signal. For an EDFA, the best possible NF is 3 dB, and therefore, the same expression is reached in a shot-noise-limited dual-quadrature receiver assuming a 100% detector quantum efficiency^15^.

In contrast to EDFAs, PSAs have a theoretical NF of 0 dB, thus amplifying the signal without excess noise^16^. A PSA that can amplify both quadratures of an optical wave requires two input waves at different wavelengths, the signal and its conjugate (idler) and a single pump. Although PSAs are available in many different configurations, the one-pump non-degenerate signal/idler configuration, which is simple to implement and amplifies both light quadratures^17^, was adopted in this work.

Due to the coherent addition of the input signal and idler by parametric amplification and incoherent addition of their noises, the output SNR of each wave after the PSA is 3 dB higher than the input, corresponding to a NF of −3 dB, which is mathematically explained in the “Methods” section. However, the overall quantum-limited NF of the PSA is still 0 dB when accounting for both the required input waves containing the same information^18^.

Due to the four-fold SNR improvement over the EDFA (which degrades the SNR by 3 dB), the capacity of the PSA is ${\it{C}}_{{\rm{PSA}}} = \frac{B}{2}\log _2\left( {1 + 4{\it{n}}_{\rm{s}}} \right)$, where the factor 1/2 is due to the loss of the SE as the signal and idler carry the same information (please refer to the Supplementary Material for a detailed derivation).

In the limit of SNR → 0, the capacity of the PSA-amplified receiver is ${\it{C}}_{{\rm{PSA}}} = 2Bn_{\rm {s}}/\ln 2$, double that of the EDFA, namely, $C_{{\rm {EDFA}}} = Bn_{\rm {s}}/\ln 2$. The ultimate sensitivity is determined by calculating the ratio of the number of photons per symbol *n*_s_ to the number of bits per symbol (C/B), which is equal to the SE (bits/s per Hz), resulting in the best possible sensitivity of 0.35 PPB for the PSA and 0.7 PPB for the EDFA. In this work, we experimentally show that PSAs can reach a black-box sensitivity of 1 PPB, whose preliminary results were presented in our previous publication^19^. Here, we extend this work by including a theoretical analysis of the achievable sensitivities with PSAs.

## Experimental setup

**Fig. 1:**
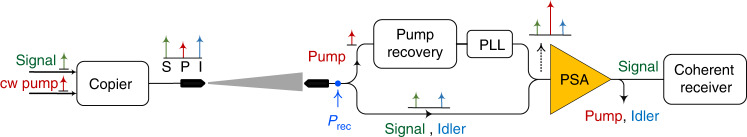
Conceptual diagram of a free-space communication link with a PSA pre-amplified coherent receiver. S signal; P pump, I Idler, PLL phase-locked-loop, PSA phase-sensitive amplifier

Figure 1 shows a conceptual diagram of a free-space optical transmission link with a PSA pre-amplified receiver. At the transmitter, a binary data stream was FEC-encoded using a code from the digital video broadcasting standard (DVB-S2), consisting of a concatenation of an inner 1/2-rate low-density parity-check code (LDPC) and an outer high-rate (0.6%) Bose–Chaudhuri–Hocquenghem (BCH) code. The data was modulated onto the signal with quadrature-phase-shift-keying (QPSK) modulation at a symbol rate of 10.52 Gbaud, resulting in a net information rate of 10.52 Gb/s. The signal was then combined with a continuous-wave pump in the copier stage to generate a conjugate idler wave, containing the same information as the signal, by four-wave mixing (FWM) in a nonlinear optical fibre. All three waves, i.e., signal, idler and pump, were then amplified by a booster amplifier (not used in our experiment) to the desired output power and launched into the free-space channel; in our case, a short 1-m free-space link was implemented in the laboratory followed by an optical attenuator to emulate the beam diffraction-induced loss in a real link. The free-space link was adopted to confirm that no additional penalty occurs when launching waves into free space. It should be noted that the NF of the copier and booster amplifier do not need to be below 3 dB. In fact, they could be substantially higher without causing any sensitivity degradation in the receiver. The reason for this is that any excess noise from the transmitter is attenuated to a level such that the quantum noise (−61 dBm at a bandwidth of 0.1 nm) dominates at the receiver due to the large link loss^18^.

The received power (*P*_rec_), defined as the total power of the signal, idler and pump after the receiver collimator, as depicted in Fig. 1, represents the black-box receiver sensitivity excluding the coupling efficiency of the collimator. The launch power determined by the booster amplifier is equally important in terms of the receiver sensitivity in an actual free-space link. It is therefore essential that the pump power corresponds to only a small fraction of the total launch power. In our case, the pump power was substantially lower than the combined signal and idler power, resulting in a nearly negligible power budget penalty.

At the receiver, the pump was separated from the signal and idler with a wavelength division multiplexer (WDM) and recovered using optical injection locking (OIL)^20^. We were able to recover a stable high-power (~1 W) pump wave at input power levels as low as −72 dBm, which is at least 12 dB lower than the received signal power level. The amplification of the pump was thus >100 dB.

An optical PLL after the pump recovery maintained a constant relative phase between the three waves for a maximum phase-sensitive gain. The signal, idler and recovered pump were then combined inside an HNLF for phase-sensitive amplification of the signal. After the PSA, the signal was filtered and detected using a standard coherent receiver and a real-time oscilloscope for subsequent off-line digital signal processing (DSP). As the idler is not needed for the detection, the receiver bandwidth requirement is the same as that of an EDFA pre-amplified receiver operating at the same symbol rate.

## Results

**Fig. 2:**
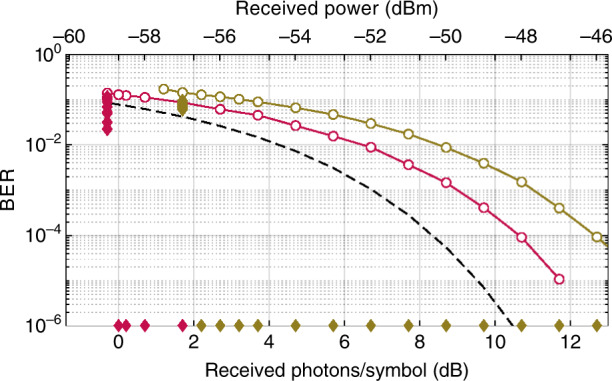
Experimental results with 10.52-Gbaud QPSK data, showing BER versus received power (also expressed in photons per symbol) before and after FEC decoding for: EDFA pre-FEC (the brown line with open circles as the measured points); EDFA post-FEC (the brown solid diamond markers); PSA pre-FEC (the red line with open circles as the measured points); PSA post-FEC (the red diamond markers). The dashed black line shows the theoretical estimate for the QPSK data for a PSA with a 0-dB noise figure. Note that the power scales represent the total receiver power, i.e., the signal, idler, and pump power, in the case of PSA

The bit error rate (BER) of the received signal was measured to evaluate the performance of the PSA pre-amplified receiver and was also compared to an EDFA pre-amplified receiver as shown in Fig. 2. The power scales include the total input power, i.e., the signal, idler, and pump waves for the PSA, and only the signal power for the EDFA. The power budget penalty caused by the presence of the pump wave in the PSA (~12 dB below the combined power of the signal and idler) was at most 0.26 dB. The pre-FEC BER in Fig. 2 shows that the PSA perform 2.5 dB better than the EDFA-based receiver, which is attributed to difference in NFs of the amplifiers, measured to be 1.2 dB for the PSA and 3.7 dB for the EDFA. No additional power penalty occurred due to the free-space propagation compared to a back-to-back scenario with only an attenuator.

The post-FEC BER was determined after FEC decoding and is also shown in Fig. 2. A coding gain of 11.8 dB was obtained at a BER = 10^−5^ for both the EDFA and PSA pre-amplified receivers. The results show that error-free (below BER = 10^−6^, limited by the memory of the real-time oscilloscope and offline processing) transmission can be achieved with a received power of 1 photon/symbol or 1 photon/information bit (PPB) (including FEC overhead) with a PSA pre-amplifier which is the best black-box sensitivity reported to date. This result is more than 3 dB better than the previously best reported sensitivity of 2.1 PPB at similar data rate and FEC^12^. In our EDFA case, an error-free performance was achieved at 1.7 PPB, 2.3 dB higher than for the PSA. The received power measurement uncertainty based on the calibrated power meter was ±0.1 dB or equivalently ±0.02 PPB. We estimated the possible sensitivity of our specific system with an ideal FEC using generalized mutual information (GMI) as 0.85 PPB. A discussion on the GMI results is presented in the Supplementary Material.

## Discussion

Importantly, PSA pre-amplification is compatible with other methods for further sensitivity improvement, e.g., power-efficient modulation formats, spatial/spectral diversity and advanced soft-decision FEC, and is straightforwardly scalable to higher bit rates as well as other wavelengths using a different nonlinear platform. It should be noted that transmitting both signal and idler results in a reduction in the SE in the optical domain. The PSA approach therefore represents a trade-off of SE versus sensitivity somewhat similar to that of PPM modulation formats. However, in contrast to PPM, because there is no need to detect the idler wave in the PSA approach, there is no particular requirement on the bandwidth versus the bit rate in the electrical domain.

**Fig. 3:**
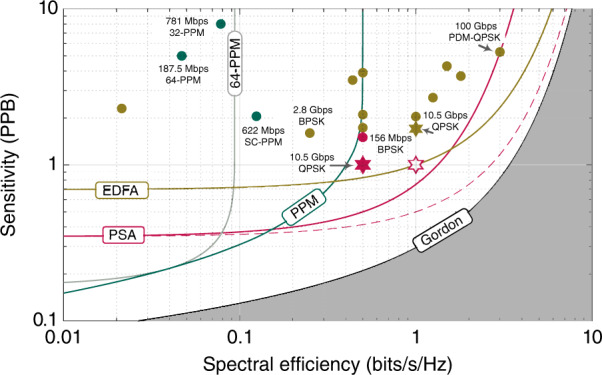
Sensitivity (photon-per-information-bit, PPB) versus spectral efficiency (bits/s/Hz) with different implementations. Theoretical curves are indicated by lines, while the experimental data are indicated with symbols. Gordon’s capacity limits for reliable transmission of information including quantum effects (black)^21^, the shaded grey area is thus fundamentally inaccessible; capacity of DQ-coherent homodyne detector with PSA pre-amplifier (red), PSA with no loss in spectral efficiency due to the idler (dashed red) and EDFA pre-amplifier (brown); envelope of all PPM capacities^3^ (green) and 64-PPM capacity (grey). Experimental sensitivity records of photon counting receivers (measured in incident PPB, i.e., the black-box sensitivity) with PPM technology at net data rates >100 Mb/s (green markers); record sensitivities of advanced modulation formats with pre-amplified coherent receivers at net data rates >100 Mb/s (brown markers), single-quadrature detector (red marker); the experimental data was extracted from the refs. ^9–14,26–28^; the PSA result presented here is denoted by a red star (red filled and unfilled), and the EDFA result is represented by a brown star

Figure 3 depicts the trade-off between SE and sensitivity for receivers used in free-space communications along with the experimental sensitivity records using these techniques. PPM is plotted as the envelope of all *m*-ary PPMs (green line) showing the best achievable sensitivity for a given SE. A specific example format, 64-PPM, is also plotted, as it is frequently used in space communications. Although PPM formats provide the best possible sensitivity at very low spectral efficiencies, they require large receiver bandwidth to achieve high information rates, which is very challenging with photon counting receivers.

Coherent receivers with PSA pre-amplifiers not only achieve a 3-dB sensitivity advantage over EDFA pre-amplifier-based receivers at low SE, but are also much more spectrally efficient than PPM formats. PSA pre-amplified coherent receivers amplify both quadratures of the signal and reach the best sensitivity among all receivers at spectral efficiencies ranging from 0.16 to 1.6 b/s/Hz. Considering the state-of-the-art signal bandwidth of 60 GHz, this corresponds (in the ideal case) to data rates varying between 9.6 and 96 Gbit/s, which is extremely relevant for future space communication systems.

The theoretical lines of the PSA and EDFA intersect as EDFAs provide better sensitivity at high spectral efficiencies (>1.6 b/s/Hz) compared to PSAs as the PSA requires transmitting a signal and idler, thus twice the bandwidth. However, for single-channel systems, as employed in space communications, the receiver bandwidth utilization is more important than the optical SE due to the unrestricted channel bandwidth and limited receiver bandwidth. In this case, the SE loss due to the idler can be ignored because at the same symbol rate, the receiver bandwidths of the PSA and EDFA pre-amplified receivers are the same. The PSA curve then shifts towards the right by 3 dB, as indicated by the red dashed line in Fig. 3. The result, if we also ignore the loss in optical SE in the experiment, is indicated by the white star.

It should be noted that the PSA achieves the same theoretical sensitivity as an unamplified ideal SQ detector with a 100% quantum efficiency and without thermal noise^21^, which is known to provide the best sensitivity among non-photon counting receivers. However, PSAs provide noise-free amplification for both signal quadratures, resulting in twice the data rate for the same receiver bandwidth. In addition, the amplification eliminates the impact of thermal noise and limited quantum efficiency, thus making it more experimentally realistic to reach the fundamental sensitivity limit.

Our experimental result (1 PPB) is ~3 dB above the theoretical sensitivity at the SE (0.5 b/s/Hz). The factors causing this penalty include the NF of the PSA (1.2 dB), implementation penalty of the QPSK transmitter and receiver (0.4 dB), DVBS2 limit (0.7 dB from Shannon limit), losses of the WDM couplers (0.2 dB), the presence of the pump power and penalty due to the phase noise added by the injection locking mechanism (0.3 dB).

Our sensitivity result is 3 dB better than the previously reported sensitivity of 2.1 PPB at similar data rate and FEC overhead^12^. When comparing to non-coherent approaches, our sensitivity is better than the important demonstrations operating above 100 Mbps while operating at more than 10 times higher data rates, as shown in Fig. 3. In short, a phase-sensitive amplifier with a coherent receiver provides the best sensitivity–data rate combination.

While this demonstration focused on the application in deep-space links, it can also be used in atmospheric links to improve the sensitivity. However, the sensitivity benefit may be lower than 3 dB depending on the turbulence strength. The effect of atmospheric turbulence on the PSA pre-amplified receiver performance was studied previously^22^.

We conclude by noting that a black-box record sensitivity of 1 PPB was demonstrated at 10.5 Gbps using a simple, spectrally efficient modulation format, enabled by the PSA and ultra-low power injection locking-based pump recovery. This sensitivity is achieved by approaching the fundamental coherent reception limit of optical signals with a novel noise-free phase-sensitive pre-amplifier. PSA pre-amplified coherent homodyne receivers are the most sensitive receivers in the practical SE range from 0.16 to 1.6 bits/s/Hz, and in comparison to solutions relying on spectrally inefficient PPM formats, they result in an order of magnitude better receiver bandwidth utilization. The fundamental advantages enable reach extension, increase of information rate and/or reduction of size of the involved optics and we believe that these results represent a significant contribution in the fields of space communication and LIDAR applications such as Earth monitoring.

## Methods

### PSA operation

Phase-sensitive amplification can be explained by the following transfer matrix:

$$\left[ {\begin{array}{*{20}{c}} {B_{\rm {s}}} \\ {B_{\rm {i}}^ \ast } \end{array}} \right] = \left( {\begin{array}{*{20}{c}} \mu & \nu \\ {\nu ^ \ast } & {\mu ^ \ast } \end{array}} \right)\left[ {\begin{array}{*{20}{c}} {A_{\rm {s}} + N_{\rm {s}}} \\ {A_{\rm {i}}^ \ast + N_{\rm {i}}^ \ast } \end{array}} \right],$$

where *A*_s_ and *A*_i_ are the input fields and *B*_s_ and *B*_i_ are the output fields of the signal and idler, respectively, *N*_s_ and $N_{\rm {i}}^ \ast$ are the uncorrelated quantum noise fields at the input waves, and *μ* and *v* are complex transfer matrix coefficients, which depend on the physical parameters of the amplifier and satisfy the following relation^23^:

$$\left| \mu \right|^2 \,-\, \left| \nu \right|^2 \,=\, 1.$$

The equations of the output fields derived from the transfer matrix are

$$\begin{array}{l}{\it{B}}_{\rm{s}} = \mu {\it{A}}_{\rm{s}} + \nu A_{\rm {i}}^ \ast + \mu N_{\rm {s}} + \nu N_{\rm {i}}^ \ast \\ B_{\rm {i}} = \nu A_{\rm {s}}^ \ast + \mu A_{\rm {i}} + \nu N_{\rm {s}}^ \ast + \mu N_{\rm {i}}\end{array}.$$

Assuming an equal power in both the input signal and idler waves, the signal gain (ignoring the noise fields) can be expressed as

$${\it{G}} = \frac{{\left| {B_{\rm {s}}} \right|^2}}{{\left| {A_{\rm {s}}} \right|^2}}\, =\, \left| {\mu + \nu } \right|^2 \,=\, \left| \mu \right|^2 \,+\, \left| \nu \right|^2 \,+\, 2\left| \mu \right|\left| \nu \right|\cos \left( \phi \right),$$

where *ϕ* = *ϕ*_s_ + *ϕ*_i_ + *ϕ*_*μ*_ − *ϕ*_*v*_ is a constant and maintained at zero experimentally. Hence, the signal gain can be written as ${\it{G}}_{{\rm{signal}}} = \left( {\left| \mu \right| + \left| \nu \right|} \right)^2$.

Since the quantum noise is uncorrelated at all frequencies, 〈*N*_s_*N*_i_〉 = 0, where $\left\langle . \right\rangle$ is the expectation operator, and the noise gain can be expressed as ${\it{G}}_{{\rm{noise}}} = \left| \mu \right|^2\, +\, \left| \nu \right|^2$.

At sufficiently high gain, $\left| \mu \right|^2 \,\approx\, \left| \nu \right|^2$^23^, we can calculate the NF of the PSA as *G*_noise_/*G*_signal_ = 1/2. Note that this is the NF of the individual waves in a PSA. However, the total NF is the sum of the individual NFs of the signal plus the idler, which is 1 or 0 dB.

### Experiment

**Fig. 4:**
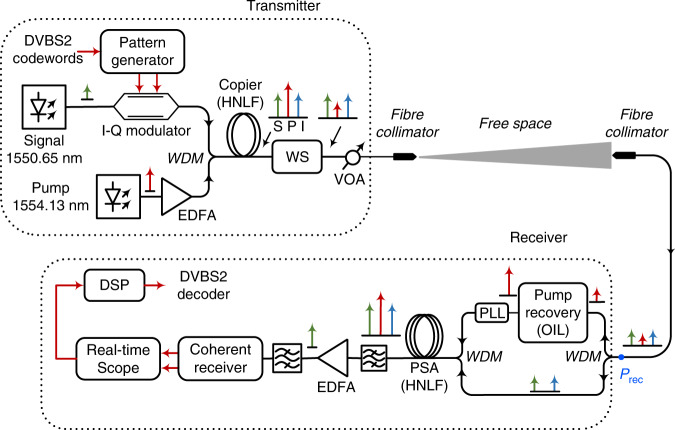
Detailed experimental set up of the free-space communication system with a PSA pre-amplified receiver. EDFA erbium-doped fibre amplifier, HNLF highly nonlinear fibre, OIL optical injection locking, WS wave shaper, VOA variable attenuator, WDM wavelength division multiplexer coupler, DVBS2 digital video broadcasting standard 2 code, PSA phase-sensitive amplifier; the electrical paths are indicated by red lines; the optical paths are indicated by black lines

Figure 4 shows the experimental setup of the transmitter and PSA pre-amplified receiver. The transmitter consists of an external cavity laser (ECL) signal laser (linewidth of 50 kHz) at 1550.65 nm, modulated using an I-Q modulator to generate 10.52-Gbaud QPSK data. The data were generated with a pattern generator (JBERT N4903) programmed with FEC encoded with a 2^15^ PRBS sequence using a DVB-S2 code, consisting of a concatenation of a 1/2-rate soft-decision LDPC code and an outer high-rate (0.6%) BCH code. The length of each code word was 64,800 bits with 10 code words in each measurement batch. The I and Q channels were modulated with the same coded bit pattern delayed by 19 symbols. The modulated signal was then combined with the fibre laser at 1554.13 nm (a linewidth of 100 Hz) as a pump to generate a conjugated idler at 1557.6 nm containing the same data as the signal via FWM in a 200-m long highly nonlinear fibre (HNLF). The loss in a free-space channel was emulated with a variable attenuator.

The fact that we transmit three waves into free space vs. only one, which is, is not essential from a power budget perspective, as a saturated booster amplifier provides the same total output power in both cases.

**Fig. 5:**
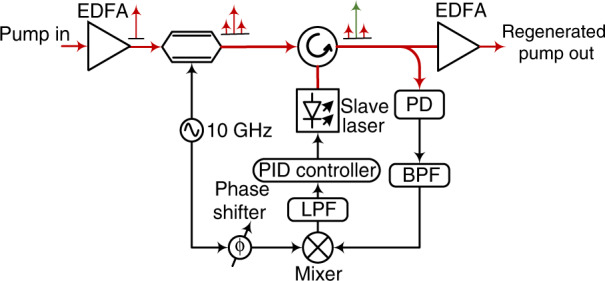
Experimental setup of optical injection locking with the PLL. PD photodetector, PID proportional integrator differentiator, BPF bandpass filter, LPF low-pass filter, EDFA erbium-doped fibre amplifier; the red lines indicate the electrical paths, and the black lines indicate the optical paths

At the receiver, the pump was separated from the signal-idler path for regeneration purposes using a WDM coupler (at a 0.1-dB loss). For the recovery of the very weak pump, we applied an EDFA pre-amplified injection-locking (IL) scheme with an electrical PLL, as shown in Fig. 5, capable of recovering a stable pump at input powers as low as −72 dBm, which was 12 dB below the measured signal plus idler power^15^.

The received signal and idler were then combined with the regenerated pump in a second WDM coupler (at a 0.1-dB loss) for phase-sensitive amplification in a cascade of 4 HNLF spools (a total length of 500 m) providing a gain of 21 dB. Cascaded and strained HNLFs with isolators between the spools were employed to increase the stimulated Brillouin scattering threshold and provide the maximum parametric gain^24^. An optical PLL after pump recovery was adopted to maintain the relative phase between the pump, signal and idler for the maximum phase-sensitive gain. The OSNR of the signal was measured using an optical spectrum analyser (OSA) and used to calculate a PSA NF of 1.2 dB. The amplified and filtered signal was then passed through an EDFA (G ~25 dB) to provide sufficient power for the receiver without notably influencing the overall NF. The signal was filtered out (the idler not being used) and detected in a coherent receiver with a free-running local oscillator (LO) laser (ECL: 50 kHz) operating close to the signal wavelength. The signal was digitized with a real-time oscilloscope at 50 GS/s over a 7-µs time duration capturing all 10 code words. The data were processed offline with a regular DSP chain consisting of IQ-imbalance compensation, frequency offset estimation, CMA equalization (seven taps) and phase estimation using blind-phase search (the code is available as an open-source code)^25^, without data-aided pre-convergence, before FEC decoding. An implementation penalty of 0.6 dB to transmit and receive the QPSK data was measured.

### OIL below a 100-pW input power

OIL with an external electrical PLL was employed to regenerate the weak pump (a minimum of −72 dBm) at the PSA receiver. Injection locking was performed using an isolator-less DFB laser isolated from external vibrations and noise. As the injection locking bandwidth depends on the injected power, locking could become unstable at low injected powers. An external electrical PLL, as shown in Fig. 5, was implemented to maintain the locking stable by constantly monitoring the locked phase of the slave, which depends on the free running frequency difference between the master and slave lasers in the locked state and correcting for the frequency shift in a feedback loop controlling the current to the laser. The PLL maintains a stable locking state at an injection power as low as −55 dBm. To regenerate the pump at even lower powers, a pre-amplifier (the EDFA) was introduced to amplify the low-power pump prior to injection locking. The noise transfer through the injection locking process is minimized by optimizing the slave input power. As injection locking is a polarization-dependent phenomenon, we used polarization-maintaining fibres in the setup and adjusted the pump to the desired state of polarization. A complete study of the noise transfer through injection locking and its dependence on the slave laser linewidth was provided in our previous work^20^. The minimum locking power demonstrated in our previous work was −65 dBm^20^. By further optimization of the system, we performed locking down to a pump power level of −72 dBm in the current experiment. The frequency-locked pump was further amplified using the high-power EDFA to provide sufficient power (~1 W) for the PSA.

## Supplementary information

Supplementary Information
