# Supplementary material for: One photon-per-bit receiver using near-noiseless phase-sensitive amplification
Source: Light Sci Appl. 2020 Sep 2;9:153. doi: 10.1038/s41377-020-00389-2 (PMC7468260; doi:10.1038/s41377-020-00389-2)
Supplement: Supplementary file 1 — Supplementary Information [file 41377_2020_389_MOESM1_ESM.pdf]

Supplementary information for

One photon-per-bit receiver using near-noiseless phase-sensitive amplification

Ravikiran Kakarla, Jochen Schröder and Peter A. Andrekson*

Photonics laboratory, Department of Microtechnology and Nanoscience (MC2), Chalmers University of Technology, Gothenburg, 412-96, Sweden.

*Corresponding author: Peter A. Andrekson, Email: peter.andrekson@chalmers.se

Capacities of PPM and coherent homodyne receivers

Capacity of PPM encoded signals

In a photon communication channel, the probability of receiving k photons per pulse is provided by the Poisson distribution [1]

$$p(k) = \frac{n_s^k e^{-n_s}}{k!} \tag{1}$$

Where n_s is the mean number of photons per pulse. The probability of receiving no photons is $p(k=0) = e^{-n_s}$ and the probability of receiving at least one photon is $p(k>0) = 1 - e^{-n_s}$

The entropy of a *m*-PPM modulated signal captured by photon counting receivers in bits/symbol is given by [2]:

$$H_{PPM} = \left(1 - e^{-n_s}\right)\log_2(m) \tag{2}$$

which results in a capacity in bit/s [2],

$$C_{PPM} = \frac{1}{T}\left(1 - e^{-n_s}\right)\frac{\log_2(m)}{m} \tag{3}$$

where T is the pulse duration, m is the number of slots of a PPM symbol (or PPM order), and mT is the symbol duration.

The spectral efficiency of PPM (bits/s/Hz) is $SE_{PPM} = \left(1 - e^{-n_s}\right)\frac{\log_2(m)}{m}$, where $\frac{\log_2(m)}{m}$ is the maximum possible spectral efficiency, and n_s is the average number of photons per symbol, which is equal to the number of photons per pulse in a PPM symbol.

For example, for 64-PPM $SE_{64-PPM} = 0.09\left(1-e^{-n_s}\right)$ corresponding to a best spectral efficiency of 0.09 b s^{-1} Hz^{-1}.

The sensitivity of the PPM format (expressed in photons per bit, PPB) is obtained by taking the ratio of photons per symbol n_s and H_{PPM} in bits per symbol. The best possible sensitivity is found by letting n_s in eq. (2) approach zero, resulting in $PPB_{\min} = 1/\log_2(m) = 0.167 PPB$ for 64-PPM.

In principle, an arbitrarily small sensitivity can be achieved by increasing the PPM order, however, at the expense of spectral efficiency. Moving from 64- to 256-PPM improves the ultimate sensitivity by 1.2 dB (to 0.125 PPB) but requires a three-fold increase in the hardware bandwidth for the same operating bit rate.

Capacity of coherent homodyne receivers

The capacity of a single quadrature (SQ) homodyne receiver in the shot noise (SN) limited case given by [1]:

$$C_{SQ-SN} = \frac{1}{2} B \log_2\left(1 + \frac{4\varepsilon S}{h\nu B}\right) \tag{4}$$

where S is the signal power, B is symbol rate and ε is the quantum efficiency of the detector. $4\varepsilon S/h\nu B$ is the signal to noise ratio (SNR). The factor ½ in front of the capacity is due to the fact that only a single quadrature is used to transmit information.

The capacity can be expressed in terms of signal photons per symbol (n_s) since the signal power $S = n_s h\nu B$

$$C_{SQ-SN} = \frac{1}{2} B \log_2\left(1 + 4\varepsilon n_s\right) \tag{5}$$

The sensitivity of a receiver is calculated from the capacity using the relation

Sensitivity $\xi = \dfrac{n_s}{C/B}$

For a SQ receiver in the SN-limit case, the sensitivity is

$$\xi_{SQ-SN} = \frac{n_s}{\frac{1}{2}B\log_2\left(1+4\varepsilon n_s\right)} \quad (6)$$

Assuming $\varepsilon = 1$, at low SNRs (in the limit $n_s \to 0$), the sensitivity becomes $\xi_{SQ-SN} \cong \frac{n_s}{\frac{1}{2}\left(4n_s\right)}\ln(2) \cong 0.35\ PPB$ which is the best possible sensitivity of a single quadrature coherent receiver.

Capacity of a dual-quadrature (DQ)/ phase-diversity coherent homodyne receiver

In order to detect both quadratures of a signal, two SQ homodyne receivers need to be employed, where the signal power is split into half using a 3-dB splitter for each of the SQ detectors. Besides 3dB splitter loss, the signal undergoes another 3-dB loss due to the SQ detection of a DQ signal in each of the SQ detectors, thus the SNR degrades by a factor of 4 compared to the SQ case, $SNR = \varepsilon S/h\nu B$ [3] and the capacity is:

$$C_{DQ-SN} = B\log_2\left(1+\frac{\varepsilon S}{h\nu B}\right) = B\log_2\left(1+\varepsilon n_s\right) \quad (7)$$

with the corresponding sensitivity in the SN limit:

$$\xi_{DQ-SN} = \frac{n_s}{B\log_2\left(1+\varepsilon n_s\right)} \quad (8)$$

Assuming $\varepsilon = 1$, the best sensitivity of DQ homodyne receiver is, $\xi_{DQ-SN} \cong 0.7\ PPB$, which is 3 dB higher than the SQ-SN limited case.

Capacity of EDFA pre-amplified DQ coherent homodyne receiver

The best sensitivity that can be achieved by homodyne detection in the SN limit is when the detector quantum efficiency is unity. However, while detectors can have quantum efficiencies up to about 90%, modern coherent detectors for telecom systems are designed for systems with optical amplifiers before the receiver and typical quantum efficiency is less than 10%. The practical sensitivity that can be achieved is thus limited. However, by employing a pre-amplifier, one can overcome the non-ideal coherent detection due to the poor quantum

efficiency of the detectors as amplified spontaneous emission noise (ASE) will dominate over detector shot noise. The capacity of a pre-amplified DQ coherent homodyne receiver is:

$$C_{\text{preamp}} = B\log_2\left(1+\frac{GS}{\frac{h\nu B}{2\varepsilon}+\frac{GF_N h\nu B}{2}}\right) \tag{9}$$

The first term in the denominator is due to the beat of local oscillator (LO) and signal shot noise and the second term is due to the beat of ASE and LO in the coherent receiver. Here G and F_N are the gain and noise figure of the amplifier respectively. In the high gain regime $G\epsilon \gg 1$, the capacity becomes:

$$C_{\text{preamp}} \cong B\log_2\left(1+\frac{2S}{F_n h\nu B}\right) \cong B\log_2\left(1+\frac{2n_s}{F_n}\right) \tag{10}$$

For an EDFA and all other phase-insensitive amplifiers, the quantum limited noise figure is 3 dB, hence the best capacity is:

$$C_{\text{EDFA}} = B\log_2\left(1+n_s\right) \tag{11}$$

The capacity of an EDFA pre-amplified DQ receiver and SN-limited DQ receiver are therefore identical as explained by Kikuchi [3]. In other words, the SNR degradation by the ASE noise acts similarly to the 3-dB splitter in the SN-limited case.

The sensitivity of an EDFA pre-amplified coherent receiver is thus the same as that of SN limited DQ homodyne receiver:

$$\xi_{EDFA-DQ} = \frac{n_s}{\log_2\left(1+n_s\right)} \tag{12}$$

and the minimum sensitivity of an EDFA pre-amplified DQ homodyne receiver is therefore $\xi_{EDFA-DQ} \cong 0.7\ PPB$.

Capacity of PSA pre-amplified DQ coherent homodyne receiver

To calculate the capacity of a PSA pre-amplified receiver in comparison with an EDFA based receiver, we consider two input waves in the EDFA as there are two input waves for the PSA. However, the information content of the two waves i.e., signal and idler are same for the PSA whereas they are independent for the EDFA as shown in the Fig. S1.

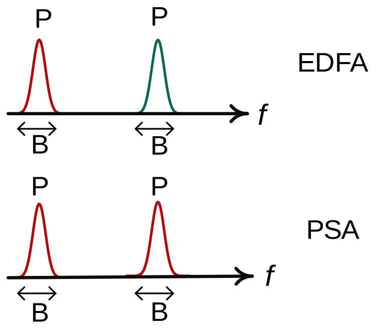

Fig. S1. Input waves of power P and bandwidth B for PSA and EDFA pre-amplifiers

In both scenarios, the available channel bandwidth is $2B$ and the power per wave is P. The total capacity is calculated as sum of capacities per wave, which is $C_{Total} = C_1 + C_2$, where C_1 and C_2 are the individual capacities of each wave.

For the EDFA, since both waves carry independent information occupying the same bandwidth with the same power, the total capacity is $C_{Total} = 2B\log_2(1+n_s)$. The capacity per wave is thus $\frac{1}{2}C_{Total} = B\log_2(1+n_s)$ as expected.

PSAs on the other hand, are known to have a noise figure of 0 dB, in reality, it is the sum of individual noise figures of modes or waves present at the input. In a two-mode PSA (our case), two input waves, signal and idler will have a noise figure of -3 dB individually. This is due to the coherent addition of input signal and its conjugated idler and incoherent addition of vacuum noise at those wavelengths in a PSA, resulting in output SNR in each wave to be twice that of input SNR or -3 dB noise figure.

The SNR in each wave is therefore 4 times higher than for the EDFA receiver i.e., $4n_s$. Hence the capacity of the signal wave is $C_1 = B\log_2(1+4n_s)$. However, the presence of the idler wave which contains the same information as the signal wave and hence has a capacity $C_2 = 0$, results in a total capacity of $C_{Total} = C_1$ and the capacity per wave is:

$$C_{PSA} = \tfrac{1}{2}C_{Total} = \tfrac{1}{2}C_1 = \tfrac{1}{2}\left(B\log_2(1+4n_s)\right) \quad (13)$$

This expression holds for DQ amplification using a 2-mode (signal & idler) PSA with DQ homodyne receiver.

The sensitivity of the PSA amplified receiver is thus:

$$\xi_{PSA} = \frac{n_s}{\frac{1}{2}\left(B\log_2\left(1+4n_s\right)\right)} \tag{14}$$

The corresponding sensitivity when $n_s \to 0$ is ξ_{PSA}=0.35 PPB

Note that sensitivity of a PSA pre-amplified DQ homodyne is same as the ideal SQ-homodyne with $\varepsilon = 1$ in the SN limited case.

Coherent intradyne receiver

Note that so far, the discussions assumed ideal homodyne detection, i.e. the local oscillator and signal carrier frequency are exactly the same frequency. Modern communication systems typically employ so-called intradyne detection, where the local oscillator is slightly detuned from the signal carrier frequency (but still well within the signal bandwidth). Such systems employ digital signal processing to compensate for the resulting frequency offset as well as phase noise resulting from the finite linewidth of local oscillator and transmitter laser. This approach can only be employed in combination with dual quadrature detection as knowledge of the full complex field is required. After digital signal processing the capacity of the intradyne receiver is equal to the dual-quadrature homodyne receiver.

Gordon's capacity of an electromagnetic wave

The maximum information carried by the electromagnetic wave is [1]:

$$C_{Gordon} = B\log_2\left(1+n_s\right) + Bn_s\log_2\left(1+\frac{1}{n_s}\right) \tag{15}$$

The sensitivity is thus:

$$\xi_{Gordon} = \frac{n_s}{\log_2\left(1+n_s\right) + n_s\log_2\left(1+\frac{1}{n_s}\right)} \tag{16}$$

When $n_s \ll 1$, the sensitivity $\xi_{Gordon} \cong \frac{1}{1+\log_2\left(1/n_s\right)}$ which approaches to zero as n_s approaches zero.

2. Generalized mutual information (GMI)

To remove the influence of the specific error-correction code on the system performance, we quantified our results using the generalized mutual information (GMI). The GMI is a measure of possible data throughput attainable for a bit-wise receiver assuming an ideal forward-error correction code [4]. Fig. S2a depicts the GMI of the received symbols, calculated from the experimental data, showing the maximum information of the received data expressed in bits per symbol. The corresponding achievable sensitivity found by taking the ratio of received power (photons/symbol) and GMI (bits/symbol) shown in Fig. S2b.

Theoretical capacities of an ideal PSA, a PSA with NF of 1.2 dB, and ideal EDFA (NF = 3 dB) is plotted in Fig. S2 a and their sensitivities in Fig . S2 b. The capacity of EDFA/PSA is plotted using the generalized eq. (10). The loss of spectral efficiency due to the idler is here not included in the PSA capacities, thus we assume a single channel transmission system limited by the receiver bandwidth, rather than by the optical spectral occupancy (see main document for discussion).

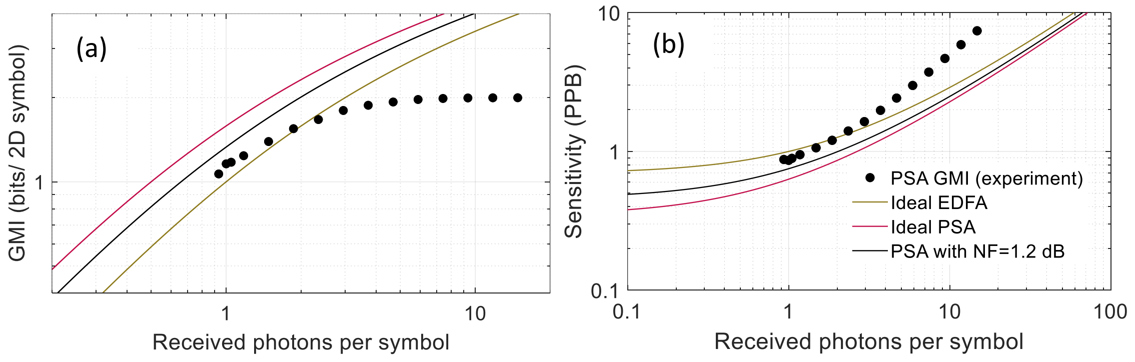

Fig. S2. (a) Generalized mutual information (GMI) of the received symbols as a function of received photons per symbol, compared with theoretical capacity curves of PSAs and EDFAs (b) Sensitivity obtained based on GMI values vs. SNR in photons per symbol, compared with corresponding theoretical sensitivities.

The lowest sensitivity based on GMI of the received data was 0.85 PPB at a GMI of 1.05 bits/symbol at -58.7 dBm power. It therefore provides a bound of sensitivity that could be achieved with a more sophisticated FEC.

References

1. Gordon, J. P. Quantum effects in Communications Systems. *Proc. IRE* **50**, 1898–1908 (1962).

2. Butman, S., Katz, J. & Lesh, J. Bandwidth Limitations on Noiseless Optical Channel Capacity. *IEEE Trans. Commun.* **30**, 1262–1264 (1982).

3. Kikuchi, K. & Tsukamoto, S. Evaluation of sensitivity of the digital coherent receiver. *J. Light. Technol.* **26**, 1817–1822 (2008).

4. Alvarado, A., Agrell, E., Lavery, D., Maher, R. & Bayvel, P. Replacing the Soft-Decision FEC Limit Paradigm in the Design of Optical Communication Systems. *J. Light. Technol.* **33**, 4338–4352 (2015).
